# Evidence on food control in charitable food assistance programs: a systematic scoping review

**DOI:** 10.1186/s13643-019-1164-8

**Published:** 2019-10-25

**Authors:** Sizwe Makhunga, Tivani Mashamba-Thompson, Mbuzeleni Hlongwa, Khumbulani Hlongwana

**Affiliations:** Department of Public Health Medicine, School of Nursing and Public Health, University of KwaZulu-Natal, Durban, South Africa

## Abstract

**Background:**

Food control is defined as a mandatory regulatory activity of enforcement aimed at ensuring that all foods during production, handling, storage, processing, and distribution are safe, wholesome, and fit for human consumption; conform to safety and quality requirements; and are honestly and accurately labeled as prescribed by law. This applies to food served by the conventional food supply chain as well as the charitable food assistance programs (CFAPs). This review sought to map the available evidence on the food control in the CFAPs globally.

**Methods:**

In order to identify the literature, we developed a series of search terms, as well as parameters for including articles to review the literature using African Index Medicus, PubMed, Google Scholar, and EBSCOhost (MEDLINE with full text, Academic search complete, MEDLINE) search engines. Articles were also searched through the “Cited by” search as well as citations included in the reference lists of included articles. We included studies reported in all languages and published from inception to 2018. We included studies if they presented evidence of the CFAPs, namely food banks, food charitable organizations (FCOs), pantries, community soup kitchens, and emergency shelters. We presented the results of our search using thematic analysis in order to reveal the emerging themes.

**Results:**

Beyond inconsistencies with the classification of the CFAPs, our study found significant knowledge gaps in crucial areas, namely food vulnerability, food traceability, vulnerability of beneficiary populations, and lack of food control. Our search yielded a total of 23 articles, which we included in the analysis. Results show that while food is the critical commodity to saving lives, if not controlled properly, it can have an adverse effect, especially on people it is meant to benefit (the vulnerable).

**Conclusion:**

With no previous comprehensive review to assess what is known about food control in the CFAPs, we undertook a scoping review, focusing on mapping the key concepts, including the main sources and types of evidence available. By drawing conclusions about the overall state of research activity and identifying research gaps and priorities in the existing literature, this study provides a baseline assessment of the CFAP research published in peer-reviewed journals from inception to 2018.

## Introduction

Although the practice of recovering and redistributing food by the charitable food assistance programs (CFAPs) has been performed for many years in many countries across the globe [1–3], little is known about food control in this sector. In this study, the CFAPs generally refer to food establishments that donate food, namely food banks, food rescue organizations, pantries, community soup kitchens, and emergency shelters. The foremost responsibility of food control is to put into effect all the applicable food laws [4–7]. This is aimed at protecting the consumer against unsafe, impure, and fraudulently presented food [5, 7]. Consumers expect protection from hazards occurring along the entire food chain, from primary producer through to consumer [5, 8]. This applies to food served by the conventional food supply chain as well as the CFAPS. In this study, we describe the food chain as the “farm-to-table” or “farm to fork” continuum.

Protection along the entire chain will only occur if all sectors in the chain operate in an integrated way and adhere to all applicable laws. The food control addresses all stages of this chain [6, 7, 9–11]. The success of such mandatory activity is largely dependent on active participation and full cooperation of all stakeholders, namely farmers, industry, and consumers. Consequently, in this study, we use the term “food control” to describe the integration of all strategies, namely regulatory, preventive, and educational strategies, aimed at protecting the food along the entire food chain. Ultimately, an ideal food control must involve the effective application of regulatory requirements, along with health and hygiene education through community outreach programs and the promotion of voluntary compliance [8, 12, 13]. Lack of food control may result in improper handling of food and the disregard for general hygiene measures [14–19]. This can result in microbiological contamination of food [15, 20–23]. In this study, we describe microbiological contamination of food as enabling pathogens to come into contact with food and, in some cases, to survive and multiply in sufficient numbers to cause illness in consumers [24, 25].

Despite the extensive food control literature in the conventional food supply chain, the current data on the CFAPs is regrettably inadequate for high-income countries (HICs) or even unavailable for low and middle-income countries (LMICs), specifically relating to the food control in the CFAPS [25–28]. In this study, we define conventional food supply chain as formal and informal food establishments and we describe the CFAPs to include food banks, food charitable organizations, food pantries, community soup kitchens, and emergency shelters. The CFAPs generally refer to food establishments that donate food. These include food banks, food rescue organizations, pantries, community soup kitchens, and emergency shelters. The scope of this study was limited to food establishments that through the process called food rescue, also called food recovery or food redistribution, divert edible wholesome food from various food enterprises to people in need, thereby contributing tremendously in the larger effort to curb the problem of food insecurity. The process is called food rescue because the food is rescued from going into the waste disposal systems [1, 3, 29–33]. These rescued foods are often referred to as “surplus” foods. Generally, the “surplus” foods are edible, but often not marketable [3, 33–36]. They comprise of food products that are at or past their “sell by” dates or are somewhat defective.

The CFAPs have earned their place as major players in the global food security system [28, 37–40]. It is for this reason that we deemed it appropriate to analyze their strengths and weaknesses in matters of food control. Our review provides a baseline analysis of the food control in the CFAPs globally based on the international guidance provided by the Food and Agriculture Organization of the United Nations (FAO) and World Health Organization (WHO). The guideline (Assuring food safety and quality: Guidelines for strengthening national food control systems) describes five key elements of a food control system [7]. These five components are (i) food law and regulation, (ii) food control management, (iii) inspection service, (iv) laboratory services, and (v) information, education, communication, and training (IECT) [7]. Thus, this review is aimed at mapping the available evidence on the food control in the CFAPs globally.

## Methods

### Approach and design

The scoping review protocol was developed and submitted to *BMC Systematic Reviews* for publication prior to commencing the systematic scoping review [41]. We conducted a scoping review following the methodology described by Arskey and O’Malley [42] and the recommendations described in the PRISMA Extension for Scoping Reviews (PRISMA-ScR) [43]: checklist and explanation (Additional file 1: Table S1). We chose this methodology as it is the most appropriate methodology for synthesizing a body of evidence that has yet to be comprehensively reviewed. Such methodology offered us an opportunity to examine the extent and nature of research related to food control of the CFAPs on Africa and the rest of the world by incorporating a range of studies with different designs (qualitative, quantitative, or mixed) and from both peer-reviewed and gray literature regardless of the quality of the study [42]. The Population, Concept, and Context (PCC) framework was also employed in this study to determine the eligibility of the research question (Additional file 2: Table S2).

### Data sources and search strategy

We conducted an initial pilot search of PubMed to ensure consistency in applying the title screening criteria (Additional file 3: Table S3). After this pilot, we undertook a more detailed search across African Index Medicus, PubMed, Google Scholar, and EBSCOhost (MEDLINE with full text, Academic search complete, MEDLINE) databases (Additional file 4: Table S4). The full search strategy (including the full search syntax) is included in Additional file 5: Table S5. We used Google Scholar to search articles through the “Cited by” search as well as citations included in the reference lists of included articles. In all search engines, we used the following search terms: charitable food assistance, food donation, food redistribution/recovery, food control, food safety, and hygiene. We used Medical Subject Headings (MeSH) terms, as well as Boolean terms (AND, OR), to separate the keywords. Gray literature search was conducted by examining non-indexed sources of evidence and creating a custom Google search to examine key websites, such as the World Health Organization (WHO), SABINET Online and World Cat Dissertations, Theses via OCLC, and Google Scholar. Publications by the Medical Research Council and Human Sciences Research Council were also reviewed. We also approached research experts on ResearchGate for any additional literature, namely thesis, conference papers/proceedings, reports, and surveys, which may not have been widely available through conventional databases.

### Eligibility criteria

#### Inclusion

The inclusion criteria are as follows:

- The CFAPs globally
- Food establishments involved in food recovery/rescue/redistribution
- The CFAPs rendering services for free (free food donation, volunteering, etc.)
- The food control in the CFAPs
- Proposed regulation in the CFAPs

#### Exclusion

The exclusion criteria are as follows:

- Charitable assistance on non-food items (e.g., clothes, equipment)
- The CFAPs rendering services for monetary gain
- CFAPs’ studies having no evidence on food control

### Process for selecting sources of evidence

We followed the outlined stages of study selection guided by the aforementioned eligibility criteria. Abstracts and full-article screening were conducted by the two independent reviewers (SM and MH) with guidance from the eligibility criteria for this study. Discrepancies between reviewers’ responses at the full-article screening stage were resolved by involving a third reviewer (KH).

### Charting the data

Data were extracted from included studies after they were thoroughly read to enable us to characterize studies included in this review. Relevant information related to the research questions was extracted using a standardized data extraction sheet. We extracted data from the following domains: author and date, study title, study setting, population, geographic setting (rural/urban), study design, type of intervention and outcomes, most relevant finding, most significant finding, study limitations and implications, and interpretation and conclusions from authors.

### Collating and reporting

We presented the results of our search using thematic analysis in order to reveal evidence of food control in the CFAPs from the included studies. In extracting themes from the eligible articles, we used QSR International’s NVivo 11 software. The full coding tree is provided as Additional file 6: Table S6. We subdivided the main theme into five components which constitute five sub-themes, namely food law and regulation, food control management, inspection service, laboratory services, and information, education, communication, and training (IECT).

## Results

The original search identified 713 peer-reviewed studies and 61 gray literatures. After removing duplicate items, the number of publications left was 579. During the title and abstract screening, we identified 541 irrelevant items. We then screened the full text of the remaining 38 papers and ended up with a final list of 23 relevant papers. The complete selection and relevant judgment process are illustrated in Additional file 7: Figure S1. Analysis of the full-article screening results showed that there was 89.47% agreement versus 56.79% expected by chance which constitutes moderate to the substantial agreement (kappa statistic = 0.77, *p* value < 0.05). In addition, McNemar’s chi-square statistic suggests that there is not a statistically significant difference in the proportions of yes/no answers by the reviewer with *p* > 0.05.

### Characteristics of included studies

Nine articles of the 23 included articles are published in peer-reviewed journals. Six of the nine are empirical study articles [12, 37, 44–47], and three are theoretical study articles [38, 39, 48]. The remaining 14 were from other sources, such as books [49–51], thesis [8, 52], policy reports [53–56], guidelines [57–60], and a manual [61]. Five of the six empirical articles were quantitative in nature [12, 44–47], and one article was qualitative in nature [37]. The three theoretical articles [38, 39, 48] were reviews. Three of the nine peer-reviewed articles were conducted in Italy [12, 44, 46], two were conducted in the USA [45, 47], and one study each was conducted in Canada [37], Spain [48], Austria [38], and Belgium [39]. Six of the nine peer-reviewed articles were focused in large cities including Asturias (Spain), Bruges and Ghent (Belgium), Florence (Italy), Vienna (Austria), and three were focused in two states, namely: Ontario (Canada) and Texas (USA). The characteristics of included studies are also shown in Additional file 8: Table S7.

### Summary of findings

Although this review focuses on the issue of food control in the CFAPs, also relevant food traceability, the vulnerability of the beneficiary population, and food vulnerability aspects will be briefly discussed, as these elements are shaping the context of the CFAPs and could have a significant impact on food control of the donated food products.

### Traceability of food

Traceability allows an investigation into whether food is considered compliant or not, locating it along the distribution chain and removing it if necessary [48]. Adopting a comprehensive and integrated farm-to-fork preventative approach to food safety recognizes that primary responsibility for supplying safe and palatable food lies with all those involved in the entire food system [8, 51]. Nine articles in this theme reported that the primary responsibility for food safety remains with the food business operator, who should guarantee it along the whole supply chain. Food should be stored appropriately, and food business operators should have procedures in place based on the Hazard Analysis Critical Control Point (HACCP) principles and produce guides to help support the correct application of safety and hygiene principles [8, 37–39, 46, 48–50, 52]. There remains a food traceability gap in CFAPs.

### The vulnerability of beneficiary populations

Thirteen articles in this theme reported that many at-risk, food-insecure individuals depend on reclaimed or rescued food, either from establishments that donate prepared and perishable foods, soup kitchens, food banks, or even field gleaning [8, 37, 39, 47, 50, 52–55, 57–59, 61]. The articles reported that this food-insecure population is at an increased risk of foodborne illness because of many factors, such as poverty, a generally poorer state of health, lack of accessible medical care, and a lower level of literacy. Among those at increased risk for certain foodborne diseases and their severe manifestations are older adults, pregnant women, young children, those with weakened immune systems (due to conditions such as AIDS, cancer, chemotherapy treatments, diabetes, or taking steroids), persons with reduced gastric acidity, and those with liver disease [8, 37, 39, 47, 50, 52–55, 57–59, 61].

### Food vulnerability

Twelve articles reported that a key aspect in the literature relating to food control in the CFAPs is that the vulnerability of the food is heightened when compared to the fresh foods with its shorter remaining life. Consequently, solid control measure is required to ensure the safety and hygiene of the foods [8, 37, 39, 45, 46, 48, 50, 51, 54, 55, 57, 59]. Three articles argued that if food donation occurs within the framework of combat of food losses, it is often donated near to the end of shelf life. Thus, the legitimate question arises whether food offered to economically deprived people is indeed still wholesome of sufficient sanitary quality and food safety is ensured [8, 39, 45].

One article reported having witnessed frozen food products contained in bulk food bins inside the food bank freezer [53]. All of the items were mixed together regardless of the product. The only exceptions were cased frozen items, which were stacked on pallets with torn boxes. The freezer was overcrowded with participants making movement quite difficult [53]. The evidence above shows that there is a gap in the food workers’ perceived factors affecting food safety and general hygiene compliance.

### Food control

As we progressively coded and compared the study articles, we found common or similar groups of concepts that were then recoded as themes. We revisited and refined concepts and categories as new insights occurred. Where necessary, we decided to group concepts and categories as sub-themes under one main theme. The following sub-themes emerged from the main theme “Food Control”: food law and regulation, food control management, inspection service, laboratory services, and information, education, communication, and training (IECT).

#### Food law and regulation

Eighteen articles in this sub-theme reported that food supplied, sold, or provided outside of the family/domestic setting is subject to food law and must be safe to eat [8, 12, 37, 39, 44–51, 53–55, 57, 59, 60]. This is regardless of whether the operation supplying or selling the food is doing so to make a profit. Food must not be “injurious to health” or “unfit for human consumption” [8, 12, 37, 39, 44–51, 53–55, 57, 59, 60]. Three articles suggested that in order to optimize the use of food losses for food donation to economically deprived persons, intense cooperation will be necessary between all stakeholders, i.e., donors, acceptors, regulators, and facilitators [37, 38, 46].

One article cited that in some of the CFAPs, workers systematically checked the expiry dates on products and inspected food packaging for evidence of threats to product safety [39]. Some CFAPs had policies or guidelines governing decisions about the safety of outdated or damaged products (e.g., not distributing baby foods that were past their expiry date, discarding boxes of cereal if the packaging inside the box had been broken). Despite the encouraging evidence presented above, there remains a gap in the administration of legislation in the CFAPs to ensure food safety and general hygiene.

#### Food control management

Twenty-three articles in this sub-theme reported that when it comes to food control management of donated food, both food donors and the CFAPs are required to comply with food safety and hygiene requirements [8, 12, 37–39, 44–61]. Nineteen articles suggest that donors are responsible for product hygiene and food safety until the moment the CFAPs accept the donated products [8, 12, 37, 39, 44–51, 53–55, 57, 59–61], although there is evidence showing that there are some CFAPs that practice food control management. For example, one article records that in some CFAPs, few workers described employing the policy, “if you wouldn’t eat it yourself, then toss it” [38]. However, there remains an integration gap between food control management by the conventional food supply chain and that of the CFAPs.

#### Information, education, communication, and training

Fourteen articles in this sub-theme argued that food workers need to fully understand that food safety training, consistent practice of hygienic food preparation practices, and regulatory inspection reports showing favorable performance histories are factors which help to protect the participants from civil and criminal liability in the good faith donation of apparently wholesome food. They further argued that good practices help to provide legal protection for the donor and help ensure the service of safe food to consumers [8, 12, 39, 45, 48–50, 53–57, 59, 61].

One article observed that when food was delivered to the food banks, workers sorted it into the categories used to structure food distribution and shelved or refrigerated the food for later use. In sorting through food donations, workers separated out items that did not fit into the standard categories and, thus, required a different process of distribution. They also separated out foods that were visibly damaged, rotting, or otherwise believed to be unsafe and foods that came in bulk, since these foods also required “special treatment” [37]. There is an information gap in how secluded unsound food is handled and disposed of.

#### Inspection service

Ten articles in this sub-theme cited that it is not always so easy to judge “acceptability” of a product for food consumption. Therefore, it is important for both acceptors and donors to agree on what is “acceptable” and under which “conditions” the food is acceptable to donate/accept, so that donated goods are still fit for consumption (not spoiled and still safe to eat) to deprived persons [8, 37, 39, 48–50, 53, 54, 57, 59].

From observations made in one of the articles, it would appear that all donated products are inspected by sight to prevent reception of compromised, damaged, or spoiled food [53]. Before accepting the donation, donor’s facilities are checked to ascertain that they are good and in hygienic conditions. The collected food is also checked that it is fit for human consumption [53]. There is a competency gap in the food workers conducting the inspection service and the involvement of the food safety regulatory authority.

#### Laboratory services

Five articles in this sub-theme reported that all donated products are inspected by sight to prevent reception of compromised, damaged, or spoiled food [8, 39, 53, 54, 61]. One article reported an observation made among the canned inventory of a large number of dents and even crushed tops [53]. There is an efficiency gap on the mechanism used by the CFAPs to ascertain food safety and general hygiene.

## Discussion

This review sought to map evidence on the food control in the CFAPs globally and identify research gaps. The FAO/WHO define food control as “the mandatory regulatory activity of the enforcement of food laws and regulations by national or local authorities to provide consumer protection and ensure that all foods during production, handling, storage, processing, and distribution are safe, wholesome and fit for human consumption; conform to safety and quality requirements; and are honestly and accurately labelled as prescribed by law” [7]. The findings of this study have helped to better underscore the existing evidence on the food control in the CFAPs globally. This review has provided evidence on the food law and regulation, food control management, inspection service, laboratory services, and information, education, communication, and training (IECT) in the CFAPs globally. To the best of our knowledge, the present study is the first systematic review of the food control in CFAPs globally. Our study findings show that the CFAPs receive food from the business industry.

Our study findings show that some of the foods received by the CFAPs are visibly damaged, rotting, or otherwise believed to be unsafe. The decision on food quality status is limited to esthetic observations and is not backed by any scientific methods in the form of a bacteriological or chemical sampling and analysis. These foods are separated out from the sound food products by the workers whose competency in food control, safety, and general hygiene is doubtful. Thus, the involvement of the food safety regulatory authorities becomes critical. It is not clear how secluded unsound food is handled and disposed of. However, research studies have shown that the vulnerability of “surplus food” is heightened when compared to the fresh foods with its shorter remaining life [8, 48, 55]. Subsequently, food control becomes the key to ensure the safety and general hygiene of the foods.

Research has shown that the charitable food assistance practice is a detour from the conventional food supply chain. Consequently, its performance in matters of food control, food safety, and general hygiene is often overlooked by the food safety regulatory authorities [39]. This is despite the evidence presenting the condition of donated food as commonly susceptible and the beneficiary populations as generally vulnerable. Generally, beneficiary populations include people at increased risk for certain foodborne diseases and their severe manifestations, namely older adults, pregnant women, young children, those with weakened immune systems (due to conditions such as AIDS, cancer, chemotherapy treatments, diabetes, or taking steroids), persons with reduced gastric acidity, and those with liver disease [52, 62–65]. The findings in other studies show that each year, as many as 600 million, or almost 1 in 10 people in the world, fall ill after consuming contaminated food. Of these, 420,000 people die, including 125,000 children under the age of 5 years [66–69]. However, research is restricted to the extent the charitable food assistance practice contributes to the above statistics. Our findings, however, show that there is widespread appeal to bring food charity practice out of the shadows, legitimize it through public education, and elevate it through public policy initiatives, in order to maximize recovery of edible surplus food [37, 38, 46].

### Study strengths and limitation

We used a rigorous and thorough search strategy for the indexed and gray literature to minimize the possibility of missing relevant literature. Further, we approached research experts in the field in order that they would provide information and contribute knowledge that was missing. As a result of this iterative approach, additional articles were included in the final thematic analysis. In addition, our full-article screening tool was piloted, resulting in increased reliability as demonstrated by the degree of agreement results. A degree of agreement showed that there were no significant differences in the screeners’ responses during full-article screening. A significant limitation is that of a Korean Journal article [70] that had to be excluded after making it through the title and abstract stage. This was after all attempts to get a Korean translator failed. As with all systematic reviews, there is a possibility that literature was missed, which may have resulted in a lack of recognition of key themes or gaps.

### Recommendations for future research

Despite the extensive food control literature in conventional food supply chain (formal and informal food establishments), the current data on charitable food assistance practice (food banks, food charitable organizations, pantries, community soup kitchens, and emergency shelters) is regrettably inadequate for high-income countries (HICs) or even unavailable for low- and middle-income countries (LMICs), specifically relating to the regulation of the CFAPs. This is despite FAO and WHO signifying that it is essential through regulatory activity of the enforcement of food laws and regulations to provide consumer protection and ensure that all foods during production, handling, storage, processing, and distribution are safe, wholesome, and fit for human consumption; conform to safety and quality requirements; and are honestly and accurately labeled as prescribed by law. We, therefore, believe that this study results will stimulate further inquiry into best ways to ensure food control in the CFAPs.

### Implications for practice and research

The findings of this scoping review have important implications for research and practice, particularly with respect to food control in the CFAPs globally. The study meant to incorporate strategies for sourcing, collection, storage, and redistribution of charitable food by the CFAPs. It intended to establish whether or not during all these processes, food control does apply to the CFAPs to ensure food safety and general hygiene management. Gaining a greater understanding of the food control in the CFAPs is imperative given the major contribution the CFAPs have in the global food security system.

## Conclusion

Although a regrettably limited literature was found in HICs, there is a paucity of data on food control in the CFAPs, particularly in LMICs. All identified articles were conducted in HICs and point to disturbing evidence of marginalization of the CFAPs in matters of food law and regulation, food control management, inspection service, laboratory services, and information, education, communication, and training (IECT). For African countries, there was not a single research article on food control, safety, and general hygiene in the CFAPs.

## Supplementary information

**Additional file 1: Table S1.** PRISMA-ScR Checklist.

**Additional file 2: Table S2.** PCC Framework.

**Additional file 3: Table S3.** Initial pilot search.

**Additional file 4: Table S4.** Database searching.

**Additional file 5: Table S5.** Full article screening results and calculations for the degree of agreement using STATA 13.

**Additional file 6: Table S6.** Full Coding Tree.

**Additional file 7: Figure S1.** PRISMA flow chart demonstrating a literature search and selection of studies.

**Additional file 8: Table S7.** Characteristics of included studies.

## Data Availability

Not applicable

## Abbreviations

AIDS: Acquired immune deficiency syndrome
FAO: Food and Agriculture Organization of the United Nations
HACCP: Hazard Analysis Critical Control Point
HICs: High-income countries
HIV/AIDS: Human immuno virus/acquired immune deficiency syndrome
IECT: Information, education, communication, and training
LMICs: Low- and middle-income countries
PRISMA: Preferred Reporting Items for Systematic Reviews and Meta-Analyses
UKZN: University of KwaZulu-Natal
WHO: World Health Organization
