# Supplementary material for: Evidence on food control in charitable food assistance programs: a systematic scoping review
Source: Syst Rev. 2019 Oct 25;8:240. doi: 10.1186/s13643-019-1164-8 (PMC6813981; doi:10.1186/s13643-019-1164-8)
Supplement: Supplementary file 2 — Additional file 2: Table S2. PCC Framework. [file 13643_2019_1164_MOESM2_ESM.docx]

**Table S2:** PCC Framework

_______________________________________________

Criteria Determnants

_______________________________________________

Population CFAPs globally

Concept Compliance

Context Food Control

_______________________________________________
