# Supplementary material for: Evidence on food control in charitable food assistance programs: a systematic scoping review
Source: Syst Rev. 2019 Oct 25;8:240. doi: 10.1186/s13643-019-1164-8 (PMC6813981; doi:10.1186/s13643-019-1164-8)
Supplement: Supplementary file 3 — Additional file 3: Table S3. Initial pilot search. [file 13643_2019_1164_MOESM3_ESM.docx]

**Table S3:** Initial pilot search

| **Population** | **Concept** | **Keywords** | **Date** | **No. found** |
| --- | --- | --- | --- | --- |
| Charitable Food Assistance System | Safety & hygiene compliance | ((("food"[MeSH Terms] OR "food"[All Fields]) AND charitable[All Fields] AND ("organizations"[MeSH Terms] OR "organizations"[All Fields])) AND ("safety"[MeSH Terms] OR "safety"[All Fields])) OR (("Appl Catal A Gen"[Journal] OR "general"[All Fields]) AND ("hygiene"[MeSH Terms] OR "hygiene"[All Fields])) | 19/02/18 | 9301 |
