# Supplementary material for: Evidence on food control in charitable food assistance programs: a systematic scoping review
Source: Syst Rev. 2019 Oct 25;8:240. doi: 10.1186/s13643-019-1164-8 (PMC6813981; doi:10.1186/s13643-019-1164-8)
Supplement: Supplementary file 4 — Additional file 4: Table S4. Database searching. [file 13643_2019_1164_MOESM4_ESM.docx]

**Table S4:** Database searching

| **Search date** | **Database** | **Keywords** | **No. of retrieved articles** | **No. of eligible titles** | **No. after canceled duplicates** |
| --- | --- | --- | --- | --- | --- |
| 20/02/18 | **Google Scholar** | Food charitable orgs, redistribution programs, hygiene, safety | 6642 | 179 – 26 | 153 |
| 21/02/18 | **Pubmed** | Food charitable orgs, surplus food, redistribution programs, hygiene, safety | 26400 | 428 – 52 | 376 |
| 22/02/18 | **Ebscohost**   - Medline - Academic search complete - MEDLINE | Food charitable orgs, surplus food, redistribution programs, hygiene, safety | 402 | 106 – 80 | 26 |
| 22/02/2018  23/02/2018  24/02/2018  25/02/2018  26/02/201827/02/2018 | **Grey Literature:**   - Thesis/dissertation - Conference proceeding - Generic - Case/technical reports - Govt. publications | Food charitable orgs, surplus food, redistribution programs, hygiene, safety | 226 | 61 – 37 | 24 |
| **TOTAL** | | | **33670** | **774 – 195** | **579** |
