# Supplementary material for: Evidence on food control in charitable food assistance programs: a systematic scoping review
Source: Syst Rev. 2019 Oct 25;8:240. doi: 10.1186/s13643-019-1164-8 (PMC6813981; doi:10.1186/s13643-019-1164-8)
Supplement: Supplementary file 5 — Additional file 5: Table S5. Full article screening results and calculations for the degree of agreement using STATA 13. [file 13643_2019_1164_MOESM5_ESM.docx]

**Table S5:** Full article screening results and calculations for the degree of agreement using STATA 13

**A Systematic Scoping Review**

**Mapping the available evidence on the food charitable organizations’ compliance with general hygiene and safety requirements in comparison with other formal and informal food establishments in Africa**

**Full Article Screening**

|  | **Author/year** | **Reviewer 1: Response** | **Reviewer 2: Response** |
| --- | --- | --- | --- |
| **1** | De Pieri et al, 2017 | 1 | 1 |
| **2** | Alexander et al, 2008 | 0 | 0 |
| **3** | Alphin, 2014 | 1 | 1 |
| **4** | Ananprakrit et al, 2017 | 1 | 1 |
| **5** | Baglioni et al, 2016 | 1 | 1 |
| **6** | Bilska et al, 2016 | 1 | 1 |
| **7** | Bonaccorsi et al, 2016 | 1 | 1 |
| **8** | Capodistrias et al, 2015 | 0 | 0 |
| **9** | Castrica et al, 2018 | 1 | 1 |
| **10** | Cheung Chi-fai, 2017 | 1 | 1 |
| **11** | Davis et al, 2014 | 0 | 0 |
| **12** | De Boeck et al, 2017 | 1 | 1 |
| **13** | Food and Environmental Hygiene Department, 2014 | 1 | 0 |
| **14** | Food Recovery Committee, 2007 | 1 | 1 |
| **15** | Food Safety Agency, 2016 | 1 | 1 |
| **16** | Foodwise.com, 2012 | 1 | 1 |
| **17** | Frasz et al, 2015 | 1 | 1 |
| **18** | Garrone et al, 2014 | 0 | 0 |
| **19** | González-Torre et al, 2016 | 1 | 1 |
| **20** | Gram-Hanssen et al, 2016 | 1 | 1 |
| **21** | Halton Region, 2011 | 1 | 1 |
| **22** | Hanssen, et al, 2015 | 1 | 1 |
| **23** | Heafz, 2003 | 1 | 1 |
| **24** | Lindberg et al, 2014 | 0 | 0 |
| **25** | Lovrenčić, 2017 | 0 | 0 |
| **26** | Mejía et al, 2015 | 1 | 0 |
| **27** | Midgleya, 2013 | 0 | 1 |
| **28** | Milicevic et al, 2016 | 1 | 1 |
| **29** | Mousa et al, 2017 | 0 | 0 |
| **30** | National environment agency, 2016 | 1 | 1 |
| **31** | NSW food authority, 2003 | 1 | 1 |
| **32** | Park, 2002 | 1 | 1 |
| **33** | Philip et al, 2017 | 1 | 1 |
| **34** | Schneider, 2013 | 0 | 1 |
| **35** | Tarasuk et al, 2005 | 0 | 0 |
| **36** | Tarasuk et al, 2009 | 0 | 0 |
| **37** | Vittuari et al, 2017 | 0 | 0 |
| **38** | Waggoner, 2004 | 1 | 1 |

1=YES; 0=NO

CALCULATIONS FOR DEGREE OF AGREEMENT USING STATA 13

kap Reviewer1Response Reviewer2Response

Expected

Agreement Agreement Kappa Std. Err. Z Prob>Z

-----------------------------------------------------------------

89.47% 56.79% 0.7564 0.1622 4.66 0.0000

. mcc Reviewer1Response Reviewer2Response

| Controls |

Cases | Exposed Unexposed | Total

-----------------+------------------------+------------

Exposed | 24 2 | 26

Unexposed | 2 10 | 12

-----------------+------------------------+------------

Total | 26 12 | 38

McNemar's chi2(1) = 0.00 Prob > chi2 = 1.0000

Exact McNemar significance probability = 1.0000

Proportion with factor

Cases .6842105

Controls .6842105 [95% Conf. Interval]

--------- --------------------

difference 0 -.1294718 .1294718

ratio 1 .8600485 1.162725

rel. diff. 0 -.3266607 .3266607

odds ratio 1 .072485 13.79597 (exact)
