# Supplementary material for: Evidence on food control in charitable food assistance programs: a systematic scoping review
Source: Syst Rev. 2019 Oct 25;8:240. doi: 10.1186/s13643-019-1164-8 (PMC6813981; doi:10.1186/s13643-019-1164-8)
Supplement: Supplementary file 6 — Additional file 6: Table S6. Full Coding Tree. [file 13643_2019_1164_MOESM6_ESM.docx]

Full Coding Tree

Nodes

Nodes\\Article information

| Name | Description | Files | References |
| --- | --- | --- | --- |
| Research article |  | 0 | 0 |
| Aims or research questions |  | 53 | 91 |
| Data analysis employed |  | 27 | 43 |
| Data Collection methods used |  | 39 | 99 |
| Definitions |  | 19 | 37 |
| Descriptions | Describing the role of FCOs | 8 | 15 |
| Focus of the study |  | 13 | 21 |
| Gaps | Call for future studies | 6 | 9 |
| Geographical setting |  | 60 | 86 |
| Limitations |  | 11 | 25 |
| Methodology |  | 18 | 31 |
| Recruitment context or study population |  | 35 | 51 |
| Study design |  | 21 | 31 |

Nodes\\Key themes

| Name | Description | Files | References |
| --- | --- | --- | --- |
| Food Control |  | 40 | 298 |
| Education, communication, and training (IECT) | Requirements such as SOPs, policies & procedures | 12 | 24 |
| Food control management | Registration, permit, certification | 9 | 14 |
| Food law & regulations | Applicable food safety and hygiene laws, regulations, policies & guidelines | 39 | 141 |
| Inspection service | In-house, external food safety & hygiene inspections/audits, food classification | 16 | 32 |
| Laboratory services and information | Implementation of food safety & hygiene statutes | 27 | 87 |
| Food hygiene and safety management |  | 34 | 464 |
| Donor exploitation | Donors abusing FCOs by using them as food waste dumps, dictating unfair terms | 9 | 51 |
| Food donors | Donating companies viz.: retail business etc. | 3 | 12 |
| Food receptors | Organizations providing food charity services e.g. NGOs, NPOs, Church organizations etc. | 8 | 10 |
| Food vulnerability | Lifespan of food | 22 | 69 |
| Protective measures | Comparing protective measures of food directed to those in need to the food prepared and served to paying consumers | 25 | 116 |
| Resources | Human, financial, logistics etc. | 23 | 127 |
| Unpredictability | Unable to challenge donors for fear of not getting sustained by them | 8 | 15 |
| Vulnerability of beneficiary populations | YOMPI | 19 | 64 |
| Food hygiene and safety principles | Food hygiene and safety requirements | 40 | 537 |
| Design of premises and facilities | The internal design and layout of food establishments | 6 | 14 |
| Food Hygiene | Condition of received food. Knowledge, Attitudes, Perceptions and Practices of donors, acceptors, and handlers. | 38 | 161 |
| Food storage and packaging | Design, construction, maintenance and general housekeeping as well as separation of food | 20 | 64 |
| Food transport | Use & maintenance of food transport, cold/heat chain maintenance | 17 | 45 |
| General housekeeping | Waste Management & Pest Control Systems | 6 | 9 |
| Health & hygiene education | Hygiene training and/or instruction and supervision | 27 | 126 |
| Labelling | Product information - directions for use and storage, warnings etc. | 17 | 67 |
| Personal Hygiene | Personal cleanliness, correct use of PPE including covering of septic wounds and reporting when unwell or diseased | 21 | 48 |
| Temperature control |  | 3 | 3 |
| Motives | What motivates food recovery and redistribution practice | 21 | 166 |
| Business | Community social upliftment, building relationships with communities | 8 | 18 |
| Environmental protection |  | 7 | 11 |
| Food loss prevention |  | 11 | 19 |
| Food security |  | 12 | 13 |
| Food waste costs | To avoid landfill site dumping costs | 4 | 7 |
| Food waste management |  | 8 | 17 |
| Historical evolution |  | 1 | 6 |
| Modern donation of food to people in need | Background of how food charity practice started | 6 | 18 |
| Politics |  | 5 | 7 |
| Public health |  | 7 | 13 |
| Social |  | 14 | 37 |
| Research gaps | Call for future studies | 6 | 9 |
| Tracking food | Ability to follow stages of food | 13 | 30 |
| Recall | Removing suspect or confirmed contaminated food | 1 | 2 |
| Traceability | Knowing where food originates and who has it | 13 | 28 |
