# Supplementary material for: Evidence on food control in charitable food assistance programs: a systematic scoping review
Source: Syst Rev. 2019 Oct 25;8:240. doi: 10.1186/s13643-019-1164-8 (PMC6813981; doi:10.1186/s13643-019-1164-8)
Supplement: Supplementary file 7 — Additional file 7: Figure S1. PRISMA flow chart demonstrating a literature search and selection of studies. [file 13643_2019_1164_MOESM7_ESM.docx]

Identification

Additional records through other sources

(n=61)

Records identified through database searching

(n=713)

Records after duplicates removed

(n=579)

(

Screening

Records excluded

(n=10)

Records screened by abstracts

(n=48)

Studies included for data content analyses

(n=23)

Studies included for quality assessment

(n=23)

Eligibility

Full-text articles assessed for eligibility

(n=38)

Full-text articles excluded with reasons (n = 15):

- Waste management strategy (n=4)
- Food loss prevention studies (n=4)
- Social impacts studies (n=2)
- Food flow (n=2)
- Food retail charity practice (n=2)
- Korean study (n=1) – No translator

Included

**Fig. S1** PRISMA flow chart demonstrating a literature search and selection of studies
