# Supplementary material for: Evidence on food control in charitable food assistance programs: a systematic scoping review
Source: Syst Rev. 2019 Oct 25;8:240. doi: 10.1186/s13643-019-1164-8 (PMC6813981; doi:10.1186/s13643-019-1164-8)
Supplement: Supplementary file 8 — Additional file 8: Table S7. Characteristics of included studies. [file 13643_2019_1164_MOESM8_ESM.docx]

**Table S8**: Characteristics of included studies

| **Author & year** | **Country/City** | **Study aim** | **Population** | **Study design** | **Research method** |
| --- | --- | --- | --- | --- | --- |
| Bonaccorsi et al (2016) | Italy | to understand whether the slow freezing, without a rapid blast chiller, represents a safe method of storage for food at the end of shelf life and can be considered an effective tool for the appropriate management of food in charitable organizations. | samples of precooked pizzas, raw poultry and raw rabbits | Experimental/analytic | Quantitative |
| Castrica, et al (2018) | Italy | to elaborate, starting from two charitable organizations selected from the head office, a checklist with an inspection score and grading, in order to assess the compliance with sanitary hygiene requirements of food banks and to make the results available to the members of the network. Finally, the checklist can represent a valuable system of self-testing and for food bank volunteers. | two food banks (hereafter CO1 and CO2) | Experimental method using inspection checklist | Quantitative |
| Davis, et al (2014) | USA | to develop transportation schedules that enable the food bank to both (i) collect food donations from local sources and (ii) to deliver food to charitable agencies. | Second Harvest Food Bank of Northwest North Carolina | Case study experimental design | Quantitative |
| De Boeck, et al (2017) | Flanders (Belgium) | to identify bottlenecks in compliance with the legal framework and implementation of food safety management, based on literature search and interviews with stakeholders | Peer reviewed scientiﬁc literature; grey literature | Systematic review | Quantitative |
| González-Torre, et al (2016) | Asturias, Spain | food waste management in the retail trade in order to improve the supply of food redistribution | the marketplaces of Asturias | Systematic review | Mixed |
| Milicevic, et al (2016) | Italy | to analyze several aspects of the food sur- plus recovery thanks to the collaboration with the Banco Alimentare Foundation Onlus and Caritas Italiana. | Food samples and the volunteers | Cross-sectional, observational, descriptive, analytic study | Quantitative method |
| Schneider, F (2013) | Austria | to show best-practice which is complemented by data on the situation of Austrian organisations dealing with the donation of food. | Austrian organisations dealing with the donation of food | Systematic review | qualitative |
| Smith, et al (2014) | Houston, Texas | to provide food safety education and improve food handling behaviors at the Ronald McDonald House in Houston, Texas. | volunteers and staﬀ | Case-control | Quantitative |
| Tarasuk, et al (2005) | Toronto, Ontario | This paper examines how the structure and function of food banks operate to facilitate the distribution of foods not marketed through the retail system | 15 food banks in and around Toronto, Ontario | Field observational & interviews | ethnographic |
| De Pieri, et al (2017) | Europe | analyses of the existing European policy on surplus  food recovery and redistribution | public administrations, food companies, non-profit organisations, and private citizens | Review | Unassigned |
| Gram-Hanssen,et al (2016) | Nordic Region | to develop a common platform for how redistribution of food can be further developed in the Nordic countries to prevent food waste without compromising food safety requirements. | food redistribution actors | survey with follow-up interviews and workshops | Mixed |
| Hanssen, et al (2015) | Nordic Region | to compile available information about laws and regulations in the Nordic countries that can be potential barriers to establishment and operation of food banks and food redistribution in general. | institutions and companies | surveys by questionnaires followed up by interviews | Quantitative |
| Food Recovery Committee; (2007) | USA | to develop guidelines for food recovery programs. | industry, consumers, academia, and federal/state/local food safety regulators | Guideline | Unassigned |
| Halton Region; (2011) | Oakville, Ontario, Canada | The materials contained in this guideline are designed to be used as a resource for training staff and volunteers | community food providers (food banks) and public health ofﬁcials | Guideline | Unassigned |
| National Environmental Agency; (2016) | Singapore | to help people comply with the law and regulations, where applicable. | organisations who intend to donate, prepare, cook and/or transport food for the needy | Guideline | Unassigned |
| Food Standards Agency; (2016) | UK | To help local authority officers make pragmatic assessments about the registration of activities such as charity food sales, food banks, community dinners for elderly people and the sales of tea and biscuits in church halls. | local authority food safety officers and organisers of community and charity food provision | Guidance | Unassigned |
| Frigo, et al (2015) | Italy | to propose correct hygiene practices to help non-proﬁt Charitable Organisations (COs) in recovering, collecting, storing and distributing food for charitable purposes in assistance of people in need, while assuring food safety | charitable organisations, companies and associations | Manual | Unassigned |
| Alphin III, Jessie; (2014) | United States | to see how 501c (3) food pantries and  food banks operate | DUMA Food Pantry-Dunn, Angier Food Pantry | Survey | Quantitative |
| CHEUNG Chi-fai; (2017) | Hong Kong | to promote donation of surplus food from the business sector | overseas legislation and policies | Review | Unassigned |
| Frasz, et al (2015) | US | report gives a summary of food rescue policies and efforts in Santa Clara County | Second Harvest Foodbank | surveys and interviews | Unassigned |
| Heafz Al Na’amah Initiative (2013) | Dubai | reduction of food losses and waste where food surplus in events is usually thrown away | donors of food such as hotels, restaurants, supermarkets and food establishments | Report | Unassigned |
| Ananprakrit, et al (2017) | Stockholm | to analyze the traceability system and the cold chain management of the food bank | Stockholm’s Stadsmission food bank | case study design | qualitative |
| Waggoner, SK (2004) | Louisiana, Mississippi, and Arkansas | to develop a strategy for preventing foodborne illness by promoting food safety practices in personnel and volunteers providing food to a vulnerable population in the Lower Mississippi Delta who utilize food recovery programs | personnel and staff of food recovery agencies | pretest-posttest knowledge design | Unassigned |
